# Exploring knowledge sharing intention of digitalization of rural intangible cultural heritage (DRICH): Integrating stimulus-organism-response (SOR) theory and social exchange theory (SET)

**DOI:** 10.1371/journal.pone.0325892

**Published:** 2025-06-13

**Authors:** Bo Zhang, Yannan Zhang

**Affiliations:** 1 College of Art, Northeastern University, Shenyang City, China; Politecnico di Milano, ITALY

## Abstract

In the digital age, knowledge sharing is gaining increasing significance, but its contribution in promoting the protection and transmission of rural intangible cultural heritage is still not fully appreciated. Based on the stimulus-organism-response (SOR) theory and the social exchange theory (SET), this study developed a theoretical model to understand the knowledge sharing intention in the digitalization of rural intangible heritage (DRICH). We examined the influence of external stimuli (i.e., heritage rewards and heritage image) on organism feedback (i.e., cultural identity, responsibility, trust, and outcome expectation) and knowledge sharing intention. A total of 363 valid samples were obtained by means of questionnaire survey and analyzed by structural equation model. The results show that: (1) Heritage rewards and heritage image, as important external stimuli, have a positive impact on cultural identity; (2) Outcome expectation, trust and responsibility positively influence knowledge sharing intention, among which outcome expectation has the strongest effect; (3) Heritage-related self-efficacy (SE) positively moderates the relationship between cultural identity and responsibility. By exploring the influence mechanism of the sharing intention in DRICH, this study provides an empirical basis for the theory and practice of the protection and inheritance of rural intangible cultural heritage.

## Introduction

As the source of intangible cultural heritage, agricultural civilization inherits the life wisdom and knowledge passed down from generation to generation [1]. Rural intangible cultural heritage is an important way to protect and inherit rural cultural resources, as well as an embodiment of rural cultural life, employment sources and knowledge dissemination [2]. It promotes and enhances cultural identity and social cohesion [3], and plays a crucial role in income generation [4], cultural self-confidence [5] and the sense of belonging [6]. At the same time, as an important measure of China’s national strategy to promote the comprehensive transformation and sustainable development of rural areas, “rural revitalization” also emphasizes the key role of digital technology in promoting the protection, inheritance and innovation of intangible cultural heritage in rural areas [7]. Rural intangible cultural heritage serves as the basis for the transformation and sustainable development of agricultural civilization in rural areas, injecting new vitality into the rural revitalization strategy and promoting economic, social, and cultural growth.

Agricultural intangible cultural heritage is the key to promoting the strategy of rural revitalization, and together with the digital heritage technology in the context of economic globalization, has jointly shaped the new pattern of cultural and economic growth in rural areas. At present, heritage digitization has gradually become an important trend in the development of cultural heritage sites [8]. Digital technology reshapes the role of users in spreading information and enhances its reach and impact [9]. As indicated by the existing studies, emerging technologies reinforce urban economies and the advantages in cultural communication. Meanwhile, they gradually demonstrate a potential in the digitization of rural intangible cultural heritages [10]. Especially, artificial intelligence and digital technology not only enhance productivity in the entertainment and cultural services sectors but also drive the growth of relevant enterprises and their capability of exportation. More importantly, they lay a foundation technically for building localized services systems in rural areas, which facilitates the adjustment to the state of competitions between urban and rural regions [10,11]. As emphasized by Camagni et al. (2023), under the context of digitization, rural development requires that the labor divisions and resources allocation between urban and rural regions are understood from the perspective of spatial restructuring [11]. Additionally, the digitization of rural cultural heritages is producing multiplier effects on regional economies, which not only optimizes the mechanisms of information flow and trading, but also enhances the attractiveness to ecological tourism and external investments [10,12]. From above, it can be seen that the digitization of cultural heritages as technology-oriented cultural practice plays a regulatory role in the urban-rural spatial structures, which is conducive to reducing the gap in development between urban and rural areas and promoting social equitability and regional coordination [13]. Among them, knowledge sharing, as an important basis for digital communication [14,15], can effectively help with problem-solving, deepen the understanding of concepts, and upgrade information processing [16], exerting a significant impact on the sustainable development of intangible cultural heritage [17,18]. Research found that although the rapid evolution of information technology and the Internet has enabled multi-channel knowledge sharing, the level of public participation remains to be improved [19]. The inheritance of rural intangible cultural heritage largely lies in the folk community, which is the important carrier of its continuity and vitality. The deep participation of the public plays an indispensable role in advancing the rejuvenation process of rural intangible cultural heritage. In addition, due to the impact of economic globalization and the lack of awareness and attention to rural areas, there is an urgent need to develop appropriate strategies and effective methods to strengthen the protection and transmission of rural intangible cultural heritage [2].

At present, relevant studies on knowledge sharing in the field of digital heritage mainly cover technological innovation of digital humanities [20], reuse of digital heritage [21], and protection and development of traditional knowledge [22], with a focus on technology realization, benefit analysis, and the prediction and impact of behavioral trends [23]. Despite the in-depth research conducted by some scholars specializing in digital heritage on the public’s willingness to share intangible heritage knowledge, there remain some notable issues. On the one hand, the main focus of existing research is on the intangible cultural heritage of a general sense, with limited studies focusing on rural heritage, especially its digital aspects. On the other hand, the existing research tends to take monotonic perspectives, and there is a lack of studies that integrate theories while considering individuals’ emotions and psychology.

To fill this gap, SOR theory is applied in this study to explore the impact of emotional factors on individual behavior intention under the context of DRICH, with the intermediary level of individual psychology and emotional state introduced [24]. Additionally, for a further investigation into the individual’s perception and emotional response, SET is introduced to reveal the important motivation behind knowledge sharing intention [25]. Finally, SET assumption is used to elucidate how the heritage-related self-efficacy (SE) influences the intention of exchange and cooperation through the internal mechanism of social exchange theory. Therefore, the perspectives of SOR theory and SET assumption are taken in this study to serve these purposes. One is to provide theoretical support for the mechanism and dynamic influence shaping public knowledge sharing intention in the context of DRICH. The other is to offer the practical direction for disseminating and protecting the cultures listed as intangible cultural heritage.

## Literature review

### Rural intangible cultural heritage

UNESCO defines intangible cultural heritage as “social practices, conceptual expressions, forms, knowledge, skills and related tools, objects, artefacts and cultural places that are considered by communities, groups and sometimes individuals to be part of their cultural heritage”. It embodies the cultural diversity of the nation and serves as an important bridge between the past and present, tradition and modernity. As a significant part of the cultural history, rural intangible cultural heritage is defined as the intangible cultural forms that have been inherited from generation to generation in rural areas and recognized by specific cultural groups [26]. It is reflected as the description of cultural life and the source of knowledge dissemination shaped in their behavior and practice over history [27]. Among them, rural areas, due to their unique cultural characteristics and authenticity, lay a solid foundation for ICH and traditional knowledge and practice. They play an essential role not only in the protection and inheritance of ICH, but also in the maintenance of cultural diversity and historical continuity in rural areas [28].

According to some studies, rural intangible cultural heritage encompasses traditional music and dance, oral traditions, social customs, rituals and festival activities, the knowledge and practice about nature and the universe, and traditional crafts [29]. Distinct from that in cities, the intangible cultural heritage existent in the rural areas is derived from long-term natural interaction and historical development. Its knowledge, beliefs, customs and norms play a vital role in rural daily life [30]. However, farmers rely heavily on this medium to disseminate information and skills among their peers. due to the influence of traditional customs and cultural norms in rural areas [26,30]. In addition, there are various problems encountered by these valuable forms of rural intangible cultural heritage. They include insufficient representation, the lack of popularity and the limited economic opportunities in the modern context [31]. Consequently, more challenges are posed to the preservation of rural intangible cultural heritage at risk of erosion and extinction [3]. Therefore, it is essential to explore public participation in the digitalization of intangible cultural heritage in rural areas and broaden knowledge sharing through social media, online education platforms, digital museums and other channels. This is conducive to the integration of intangible cultural heritage resources with modern industry, rural economic and cultural development, as well as rural revitalization and sustainable agricultural development.

### Digitization of rural intangible cultural heritage

**Fig 1:**
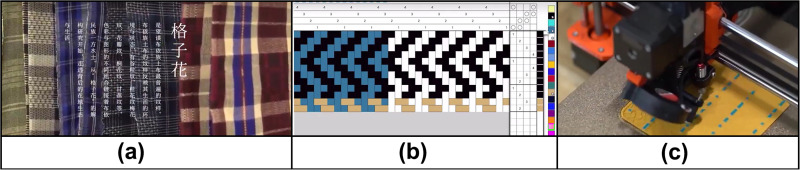
Digitalization of traditional clothing pattern conversion on Xiaohongshu platform. (a) Traditional checkered patterns (b) Digital coding and translation of checkered pattern (c) Digital model-based execution of printing and weaving.

Merriam-Webster defines digitization as “the process of transforming something into a digital form”. In the field of cultural heritage, “digitalization” refers to the integration of digital technology into daily life and the transformation of digital information into heritage [32]. Digitalization of intangible cultural heritage is the use of audio-visual records, photos and other materials to generate digital resources for preserving and disseminating community practice, knowledge, customs and related cultural elements [33–35]. This study adopts the common connotation of the digitalization of intangible cultural heritage in existing research. On this basis, the digitalization of rural intangible cultural heritage (DRICH) is defined as a digital resource used for the transformation of rural intangible cultural heritage into audio, video records, photos or other temporary materials through digital technology. Created in the course of development and practice, these resources record and preserve the practice, knowledge, customs, skills and related tools and cultural space of rural community members (Fig 1).

Public participation and wide dissemination are crucial for the development of rural intangible cultural heritage [36]. According to some studies, DRICH can improve the public’s understanding and cognition [26], with opportunities created for interaction between the dissemination of it and the public [3,37]. Specifically, the public uploads and shares intangible cultural heritage information through websites, microblogs, WeChat and other media. Thus, the visibility and influence of knowledge sharing and interaction can be enhanced [19]. In addition, from the perspective of urban-rural differences, the backwardness in popularizing technologies in rural areas results in a relative disadvantage in economic development and the inheritance of cultural heritages across these areas [11]. To reduce this gap while promoting balanced regional development, it is imperative to give full play to the popularization of digital technologies in rural areas. This is conducive to recording, preserving, disseminating and innovating on intangible agricultural heritages, which enhances their exposure and accessibility under the context of globalization. This provides a new pathway to creating economic and social values for rural regions [10,38].This is beneficial in enhancing the potential of rural cultural heritage, promoting the long-term sustainability of rural cultural practices [38,39], and facilitating the socialization and knowledge exchange and sharing of the public [40].

### Stimulus-organism-response (SOR) theory

The stimulus-organism-response (SOR) theory was originally proposed by Mehrabian and Russell [24]. At present, the SOR theory is mainly applied to research in the field of behavioral science, and has been explored in the context of heritage tourism such as return visit intention and word-of-mouth intention [41]. This theory assumes that the external environment, as a stimulus, can affect organism and behavior response, and emphasizes the role and influence relationship among stimulus, organism feedback and behavior [42]. In this theory, stimulus refers to the external factors that affect the decision-making behavior of an organism [43], Organism feedback refers to the internal structure and process between external stimulus and individual response behaviors [44], which covers emotional and cognitive responses, including perception, experience and evaluation [45]; Response refers to how organisms respond to stimulus, and some scholars also understand response as the result of decisions made by organisms in the face of changing stimulus [44].

In the existing studies, the SOR framework has been adopted to investigate the intention of knowledge sharing under the context of digital humanities. They involve various aspects such as incentive mechanism, trust construction and cognitive evaluation, for discussion about the interaction between these variables [46–48]. Among them, heritage rewards and heritage image can be taken as external environmental factors. On this basis, the individual emotions or psychological reactions related to rural cultural heritage can be stimulated by arousing individual emotional identity or reward expectation for cultural heritage [46,47]. As the organism in SOR theory, cultural identity, outcome expectation, responsibility and trust can be used to explore individual psychological and emotional feedback on the protection and inheritance of rural cultural heritage [48–51]. In this process, cultural identity is conducive to gaining insights into how individuals perceive and evaluate external stimuli, and to revealing the exact impact of organism’s psychological cognition and emotional expectations on organism’s feedback [52]. Knowledge-sharing intention is the final response made by organism to external stimuli and organism feedback. It reflects the comprehensive response made by individuals to stimuli and their own psychological state [46].

To sum up, the SOR framework is applicable to demonstrate the mechanism behind the knowledge-sharing intentions related to DRICH. This is because it associates external stimuli with individual emotional and psychological feedback. Thus, the motivation and mechanism of knowledge sharing in cultural heritage protection can be understood. Also, a theoretical support is provided for relevant research and practice.

### Social exchange theory (SET)

Social exchange theory (SET), as an important theoretical model for understanding individual behavior, plays an significant role in anthropology [53], social psychology [54–56], as well as sociology [57] and other disciplines. SET not only provides a powerful tool for analyzing and understanding the individual’s behavioral motivation and interaction pattern in social interaction, but also reveals the deep dynamic mechanism of individual social behavior. By exploring SET in depth, it is helpful to understand the complexity of social interaction and the deep driving force of interaction pattern. Hamilton & Alexander (2013) pointed out that in the assumption of SET, interaction and cooperation among individuals is the driving force of value creation [25]. Individuals can achieve value innovation and growth through the exchange and sharing of resources [58]. In addition, SET also emphasizes the important role of key factors such as trust, benefit, outcome expectation, responsibility and sense of identity in shaping individual behavior and social interaction. Outcome expectation is a social psychological phenomenon. When an individual gains benefits from social exchange, the individual will expect and tend to respond to the other party in the social exchange relationship through kindness and help behavior [59–61], and together with trust [57,62], as a variable, play an important role in the process of social exchange. Mitchell & Reid (2001) also pointed out that outcome expectation, responsibility and sense of identity are key factors to promote public interaction and exchange [63]. Among them, the sense of identity has a significant promoting effect on the supportive behavior of individuals [64,65]. Therefore, The SET is applicable to explain what motivates the public to share knowledge related to DRICH. Also, it is suitable to reveal the interaction between different variables such as cultural identity, trust, outcome expectation and responsibility. Thus, crucial theoretical support is provided to demonstrate the social-psychological mechanisms behind the sharing of knowledge related to DRICH.

## Hypothesis development

### Cultural identity, heritage rewards, and heritage image

In the field of intangible cultural heritage, cultural identity is defined as “the identification of cultural values and identities based on ICH” [66]. This perspective is important for exploring the development mechanism of ICH as well as the integration of a country or region’s special social memory and value identification system [66,67]. In rural areas, cultural identity is shaped mainly through the traditional knowledge and customs inherited from generation to generation. These factors jointly contribute to the understanding and value identity of intangible cultural heritage in rural society [3,26]. There is a close correlation between the cultural identity of rural residents to intangible cultural heritage and their identity in a specific social group. This is reflected by their role in the rural community [68]. Some studies believe that cultural identity is related to an individual’s perceived risk level [69]. Kranz & Goedderz (2020) further points out that cultural identity is closely related to an individual’s cultural perception, understanding and experience [70] Therefore, individuals’ shared cognition of intangible cultural heritage, based on cultural identity, can guide emotional and behavioral responses to promote the positive development of related activities [71].

Heritage rewards are defined as a strategy to motivate individuals or teams to achieve goals [72]. Cone (1989) pointed out that effective rewards can enhance the motivation of individual target behaviors [73]. It takes the form of both material and non-material rewards [74]. In this study, “ heritage rewards “ refer to the material and intangible benefits obtained by the public in the process of knowledge sharing of rural intangible cultural heritage. Specifically, individual attitudes will react based on economic interests and value judgments to express the degree of identification with a certain group or social norm. If the feedback perceived by the public is positive, supportive behavior will arise [75–77]. Once the public interest needs are met, their attitudes and behaviors will be further reinforced [78]. Similarly, several studies have emphasized that both economic and non-economic factors can act as key drivers of public support and recognition. [79]. In addition, individuals’ cultural identity can be enhanced by the values and benefits associated with the digitalization of intangible cultural heritage [80].

Based on the above analysis, the following hypothesis is proposed in this study:

**Hypothesis (H1): In the context of DRICH, individuals’ heritage rewards have a positive effect on cultural identity.**

In the field of cultural heritage, heritage image originally refers to the “temporal dimension of tourists’ impression of cultural heritage sites” [81]. To digitalization of rural intangible cultural heritage, it is worth extending this concept to the public’s cognitive experience of rural intangible cultural heritage in the digital environment. Also, it is equally important to extend the role of digital technology during the dissemination, acceptance and memory formation of intangible cultural heritage. Some studies have pointed out that taking heritage image as tourists’ initial perception of cultural heritage [82] is a crucial reference means to understand the characteristics of things [83]. In addition, heritage image is of great significance to individuals, and there is an influence relationship between it and cultural identity [84,85]. Chen et al. (2020) [86] further emphasized the predictive relationship between heritage image and cultural identity, discussing its positive impact in different scenarios.

Based on the above analysis, the following hypothesis is proposed in this study:

**Hypothesis (H2): In the context of DRICH, individuals’ heritage image has a positive impact on cultural identity.**

### Outcome expectation, responsibility, trust, and cultural identity

Bandura (1997) and Compeau& Higgins (1995) pointed out that outcome expectation, as a cognitive behavior, can reflect individuals’ subjective evaluation of the expected results of their own behavior [87,88]. In this study, outcome expectation is defined as the public’s subjective assessment of the potential outcomes resulting from the motivation to participate in DRICH. In this process, individuals will evaluate and determine their behavior path based on past experience, existing resources and conditions, and predictions about future environments. Among them, the expected outcome of self-interest and individuals’ perception of their own abilities and environment will have an important impact on behavioral decision-making [89,90]. Meanwhile, there are also studies that regard cultural identity and outcome expectation as two independent constructs to discuss their independent role and mutual influence in individual decision-making process [91], and how individual variables can shape outcome expectation [92,93]. Some scholars further elaborated that cultural identity positively influences outcome expectations [94,95]. In addition, the public’s expectation and willingness to buy products can be improved by the cultural identity of intangible cultural heritage, according to the commodity theory [96,97].

Based on the above analysis, the following hypothesis is proposed in this study:

**Hypothesis (H3): In the context of DRICH, individuals’ cultural identity has a positive impact on outcome expectation.**

As an emotional and cognitive response, trust can influence people’s value judgment [98]. In this study, trust is defined as the public’s trust in the reliability, transparency, and effectiveness of DRICH. Trust, as an important factor in social relations, also has a positive impact on social relations [99]. It is found that lack of trust will affect the behavioral persistence of individuals in the process of social exchange [100]. In addition, knowledge level also affects the degree to which individuals trust information sources [101]. Moreover, cultural identity is an important way for individuals to build trust [68], and a high level of cultural identity can significantly improve an individual’s trust in others [102], resulting in a higher level of commitment to the organization and its goals [103]. Besides, brand awareness and trust are reinforced by the sense of identity of intangible cultural heritage [104]. When individuals consider themselves part of a group, they feel a stronger sense of belonging and cultural commonality, increasing their trust in other members of the group.

Based on the above analysis, the following hypothesis is proposed in this study:

**Hypothesis (H4): In the context of DRICH, individuals’ cultural identity has a positive impact on trust.**

In this study, responsibility refers to the various cultural and moral duties individuals assume when sharing knowledge related to DRICH. Some studies have pointed out that responsibility is an important factor of organism [51], and individual attitude and belief are important components of responsibility [105]. Identity, as a prerequisite for responsibility [106,107], the higher its level, the more likely it is to stimulate the individual’s sense of responsibility for the protection of resources such as culture [108,109]. Furthermore, Lee (2011) also supported this view, arguing that cultural identity has a positive impact on resource conservation attitudes, including responsibility [108]. Moreover, it has been demonstrated in some studies that the public’s cultural identity is crucial for enhancing their sense of responsibility in the context of intangible cultural heritage [110,111].

Based on the above analysis, the following hypothesis is proposed in this study:

**Hypothesis (H5): In the context of DRICH, individuals’ cultural identity has a positive impact on responsibility.**

### The moderating role of heritage-related self-efficacy (SE)

Self-efficacy refers to an individual’s belief in his or her ability to perform a particular task, achieve a goal, or perform effectively [112]. Albert Bandura (1997) pointed out that it emphasizes individuals’ confidence in their own skills, ability to cope with challenges and achieve expected results [87]. Self-efficacy can have an impact on an individual’s learning and motivation through purposeful planning and systematic interventions [113] and is a key intrinsic factor driving human motivation, emotion and behavior [114]. Moreover, self-efficacy can strengthen the connection between local identity and social network [115], as well as help construct the internal connection between sense of identity and sense of responsibility [111]. Under the context of intangible cultural heritage, self-efficacy also exerts a positive regulatory effect between the sense of responsibility and public participation [111].

Based on the above analysis, the following hypothesis is proposed in this study:

**Hypothesis (H5a): In the context of DRICH, individuals’ heritage-related self-efficacy has a positive influence on the relationship between cultural identity and responsibility.**

### Knowledge sharing intention

Dyer and Nobeoka (2000) believe that knowledge sharing can promote the flow of information, improve the learning and innovation ability of organizations, and improve the efficiency of achieving preset goals [116]. Effective knowledge and information sharing can lead to competitive advantages [117] and the realization of established visions [116,118]. In this study, knowledge-sharing intention is defined as the active exchange and dissemination of knowledge, information and experience related to rural intangible cultural heritage by the public, aimed at protecting and inheriting DRICH.

Research found that knowledge sharing intention is closely related to experience level [119]. According to the SET, responsibility not only constitutes the basic condition of an organism’s behavior [120], but also plays a positive role in promoting sustainable development and behavioral decision-making of a community [121]. At the same time, some studies have pointed out that there is a significant correlation between responsibility and knowledge sharing intention [122], and responsibility plays an important role in promoting knowledge sharing intention and behavior [123,124]. This research result was further confirmed by Assiouras et al. (2019), who pointed out that individuals with a strong sense of responsibility are more likely to show positive knowledge and information sharing intention and related protective behaviors [125]. especially in the context of intangible cultural heritage [110,111].

Based on the above analysis, the following hypothesis is proposed in this study:

**Hypothesis (H6): In the context of DRICH, individuals’ responsibility has a positive impact on knowledge sharing intention**

Trust can not only enhance the intention and action of individuals to share knowledge [126], but also significantly improve the quality and efficiency of knowledge sharing [74]. Specifically, the establishment of trust can enhance the initiative of individuals to participate in sharing, which further translate into higher quality content sharing and more efficient information exchange processes. Meanwhile, knowledge sharing intention is also significantly affected by differences in trust levels [127]. Regarding intangible cultural heritage, a high level of trust is conducive to enhancing the enthusiasm of the public about intangible cultural heritage participation and exchange [128,129]. Hinds & Pfeffer (2003) further pointed out that individuals are more willing to share knowledge in highly trusted environments [130]. In addition, cognition-based trust also plays a positive role in promoting knowledge sharing intention [131]. It can be seen that trust not only strengthens the social exchange relationship between individuals, but also stimulates the motivation of individuals to share knowledge [132].

Based on the above analysis, the following hypothesis is proposed in this study:

**Hypothesis (H7): In the context of DRICH, individuals’ trust has a positive effect on knowledge sharing intention**

Bock and Kim (2002) believe that individual’s rationality and self-interest tendency are important factors affecting his behavioral intention [133]. Relevant studies have shown that there is a potential influence relationship between outcome expectation and knowledge sharing intention [89,134]. In the context of knowledge sharing, individuals’ positive perception of outcome expectation is an important factor to stimulate their willingness to share knowledge [133]. In the field of rural intangible cultural heritage, individuals’ expectation of the results exerts influence not only on their behavioral intention and performance, but also on their enthusiasm for participation and communication [128]. Individual knowledge sharing intention is driven by a variety of positive expectations, including self-satisfaction and social approval [135,136]. Kankanhalli, Tan and Wei (2005) further pointed out that individuals also expect their sharing behavior to bring social returns [137]. This expectation further solidifies their willingness to share knowledge and enhances their motivation and willingness to participate in relevant activities [89].

Based on the above analysis, the following hypothesis is proposed in this study:

**Hypothesis (H8): In the context of DRICH, individuals’ outcome expectation has a positive effect on knowledge sharing intention.**

**Fig 2:**
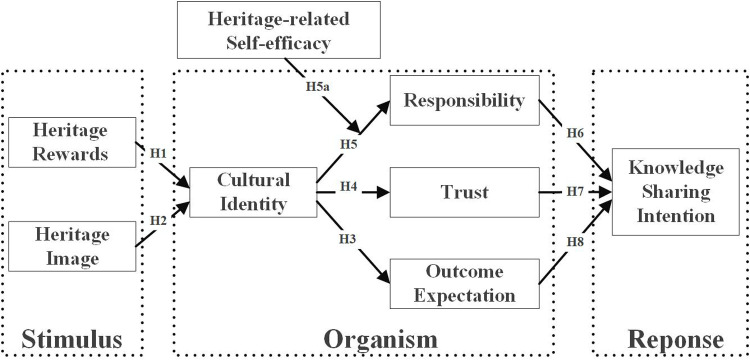
The conceptual framework of the research.

Based on the above discussion, this paper takes the SOR theory and SET assumption as the research framework, integrates a conceptual theoretical model, and shows the relationship between various variables in this study (Fig 2).

## Methods

### Measurement scales

Ethics Committee approval was obtained from the Ethics Committee of Northeast University (China) in writing (Ethics approval number: S-168/2024). The research instrument, which includes live streaming video cases and measurement scales, has been approved by the Ethics Committee of Northeast University.

The video case was obtained by intercepting the video material on the app platform. The criteria applied for video material included the relevance to the research topic and the comprehensibility of the content. On this basis, the video material was redesigned to obtain the video case of the study. Furthermore, the video case of this study was distributed in a small range to establish whether the video can be fully understood by the subjects. After the adjustments made based on the suggestions, the final video case was obtained, with a duration of 2 minutes and 43 seconds.

The measurement scales of this study were derived from the proven-mature English scale, and the current questions were designed using the original questionnaire. Among them, the items of the scales were presented in the form of statement, with adjustment made to the readability of the language. The content of the Chinese scale was created to meet the needs upon the examination and approval of three peer experts and the small-scale pre-test and adjustment. A 5-level Likert scale was used in this study, with values ranging from 1 (strongly disagree) to 5 (strongly agree). Respondents chose a scale of 1–5 that corresponded to how strongly they agreed with the statement, based on their own feelings and experiences.

In this study, 4 items were used to measure Heritage Rewards (HR), which were developed based on Boshoff & Allen (2000) [138], 5 items were used to measure Heritage image(HI), which were developed based on Tasci et al. (2022) [139], 4 items were used to measure Cultural identity (CI), which were developed based on Zhang et al. (2020) [140], 3 items were used to measure Outcome expectation(OE),which were developed based on Lin & Hsu (2015) [90], Trust (TRU)was measured using 3 items [141], Responsibility(RES) was measured using 3 items [111], Knowledge sharing intention(KSI) was measured using 4 items, which were developed based on Chuang et al.(2015) [142], and individuals’ heritage-related self-efficacy (SE) was measured using 3 items, developed based on Yang et al.(2022) [111]. The variables of this study were controlled through the above items. (Table 1)

**Table 1:** Modified scale measures.

Reference	Measure	Scale Items
Boshoff & Allen (2000)	Heritage Rewards (HR)	I will be rewarded for my excellence in digitization of intangible cultural information in agricultural systems.
		I will be rewarded for the good evaluation I receive in matters related to the digitization of intangible cultural information in agricultural systems.
		I will be rewarded for effectively solving problems in digitization of intangible cultural information in agricultural systems.
		I am rewarded for handling problems in digitization of intangible cultural information in agricultural systems appropriately.
Tasci et al. (2022)	Heritage Image (HI)	Intangible heritage digitalization related projects in agricultural systems have better appeal.
		Intangible heritage digitalization related projects in agricultural systems can be exciting.
		Intangible heritage digitalization related projects in agricultural systems have diverse content and forms.
		Intangible heritage digitalization related projects in agricultural systems have many fascinating aspects.
		Intangible heritage digitalization related projects in agricultural systems have friendly practitioners.
Zhang et al. (2020)	Cultural identity (CI)	Digitization of intangible cultural in agricultural systems represent rich cultural value.
		Digitization of intangible cultural in agricultural systems can preserve and transmit traditional culture.
		Digitization of intangible cultural in agricultural systems can leave a strong cultural impression on me.
		Digitization of intangible cultural in agricultural systems can help me express my cultural identity and values.
Lin & Hsu (2015)	Outcome expectation (OE)	I believe that participating in projects related to the digitization of intangible cultural heritage in agricultural systems will provide a profound sense of personal fulfillment.
		I believe that participating in projects related to the digitization of intangible cultural heritage in agricultural systems, I can have a hopeful and sustainable future.
		I believe that participating in projects related to the digitization of intangible cultural heritage in agricultural systems, I can be a lover of intangible cultural heritage.
Yang et al. (2021)	Trust (TRU)	I trust the perceived value and reliability of intangible cultural heritage digital projects in agricultural systems.
		I trust the information and data security of intangible cultural heritage digital projects in agricultural systems.
		I trust that digital agriculture and intangible cultural heritage will provide excellent service capabilities.
Yang et al. (2022)	Responsibility (RES)	I am willing to complete the tasks related to the digitalization of agricultural intangible heritage.
		I am willing to accomplish the goals I set for myself in the digitalization of agricultural intangible heritage.
		I am willing to fulfill the responsibility of conservation and inheritance related to the digitalization of agricultural intangible heritage.
Chuang et al. (2015)	Knowledge sharing intention (KSI)	I am inclined to share relevant knowledge about agricultural intangible cultural heritage digitization in the near future.
		I am likely to share knowledge related to agricultural intangible cultural heritage digitization with my colleagues in the future.
		I will try to share knowledge related to agricultural intangible cultural heritage digitization with my colleagues.
		I plan to share knowledge related to agricultural intangible cultural heritage digitization with my colleagues.
Yang et al. (2022)	Heritage-related Self-efficacy (SE)	I am willing to share knowledge about digital agriculture and intangible cultural heritage with others.
		I am willing to engage in effective interactions with others regarding information on digital agriculture and intangible cultural heritage.
		I am willing to participate in the relevant activities in the development of intangible cultural heritage digitalization in agricultural systems.

### Data collection

The questionnaire was developed and designed for the purpose of this study. According to the model design and research theme, sample data were collected in this study through questionnaire survey. The target population of this study included the individuals over the age of 18 who may potentially engage with DRICH, along with other relevant stakeholders. During questionnaire collection, the subjects were asked to fill in the questionnaire after watching the video case. Furthermore, data acquisition was performed both online and offline to include a wider range of groups, given the activities and preferences of different groups. Among them, online data were collected through the Wenjuanxing platform (https://www.wjx.cn/), while offline data were collected through paper questionnaire. Before the formal investigation, questionnaires were first distributed in a small range, and the quality assessment and modification of research tools were carried out according to the feedback results. The survey was conducted from April 15 to May 13, 2024. Besides, Before the survey, we introduced the research objectives to the participants and obtained their written consent and made sure to watch the background video of the study before answering the questionnaire (This video was developed and produced based on the purpose of the study, and watching this video can help subjects further understand the background information necessary to fill in the questionnaire).

### Data analysis

To effectively evaluate and verify the theory and research hypotheses, this study applied IBM SPSS 26.0 and AMOS 24.0 software to analyze the data through descriptive statistics, confirmatory factor analysis (CFA), and structural equation modeling (SEM).

## Results

### Descriptive analysis

Descriptive statistical results show, a total of 363 valid questionnaires collected in this study met the minimum sample size required by the structural equation model [143]. Among them, 35% were men and 65% were women, and the majority of respondents are in the 18–25 age group, making up 44.9%. The highest education level of the respondents is a college degree, accounting for 53.4%, the highest monthly income of the respondents was 2000–5000 RMB, accounting for 46% (Table 2).

**Table 2:** Participants’ profile.

Measure	Questionnaire items	Frequency (N = 363)	Percentage (%)
Gender	Male	127	35.0
	Female	236	65.0
Age (years)	18- 25	152	41.9
	26-35	90	24.8
	36-45	46	12.7
	Over 46	75	20.7
Education	Secondary education	110	30.3
	University education	194	53.4
	Master’s or PhD	59	16.3
Monthly Income (RMB)	Under 2000	103	28.4
	2000-5000	167	46.0
	5000-8000	59	16.3
	Over 8000	34	9.4
Total		363	100.0

### Assessing the measurement model

Before evaluating the research hypothesis, the measurement model was analyzed based on the reliability and validity results. Table 3 shows that all dimensions in this study are reliable, and the Cronbach’s α value of each dimension ranges from 0.794 to 0.907, which conforms to Nunnally (1978)’s suggestion that Cronbach’s α value is greater than 0.7 [144]. Meanwhile, the component reliability (CR) value of this study is between 0.799 and 0.908, greater than 0.7, and the AVE value of each dimension is greater than 0.5 [145], between 0.514 and 0.745. In addition, Table 4 shows the relationship between differential validity and correlation coefficient in the research model. According to the AVE square root of all dimensions proposed by Fornell, C., & Larcker (1981) [145], the AVE square root of all dimensions is greater than the differential validity standard of the correlation coefficient. The AVE square root of all dimensions in this study is between 0.717–0.863, which is greater than the correlation coefficient of all dimensions, reflecting the good differential validity of this study. Meanwhile, all factor loads in this study are in line with the index of no less than 0.5 [146], ranging from 0.657 to 0.877. Finally, Table 5 shows 7 fit indexes of the measurement model, including CMIN = 400.110; The CMIN/df = 1.146; AGFI (adjusted goodness of fit index) = 0.914; TLI (Tuck-Lewis index) = 0.989; CFI(comparative fit index) = 0.990; GFI(Goodness of Fit Index) = 0.931; RMSEA (root mean square error of approximation) = 0.020. All of them are in line with the recommended thresholds for the indicators of the fit degree model [147,148], which means that the measurement model has a good fit.

**Table 3:** Results of validity and reliability analysis.

Latent Variable	Measure Variable	Mean	Std. Dev	Factor Loading	Cronbach’s α	SMC	CR	AVE
HR	HR1	4.110	.605	.703	.808	.494	.809	.514
	HR2			.675		.456		
	HR3			.726		.527		
	HR4			.761		.579		
HI	HI1	4.037	.742	.830	.882	.689	.885	.608
	HI2			.692		.479		
	HI3			.854		.729		
	HI4			.843		.711		
	HI5			.657		.432		
CI	CI1	3.760	.892	.820	.844	.672	.846	.579
	CI2			.772		.596		
	CI3			.701		.491		
	CI4			.745		.555		
RES	RES1	3.764	.959	.723	.799	.523	.802	.575
	RES2			.736		.542		
	RES3			.812		.659		
TRU	TRU1	3.719	.992	.779	.798	.607	.799	.570
	TRU2			.744		.554		
	TRU3			.742		.551		
OE	OE1	3.799	.870	.828	.830	.686	.832	.624
	OE2			.725		.526		
	OE3			.813		.661		
KSI	KSI1	3.733	.989	.877	.907	.769	.908	.711
	KSI2			.846		.716		
	KSI3			.814		.663		
	KSI4			.835		.697		
SE	SE1	3.184	1.192	.872	.896	.760	.898	.745
	SE2			.852		.726		
	SE3			.865		.748		

**Table 4:** Discriminate validity and correlations of the research model.

	HR	HI	CI	RES	TRU	OE	KSI	SE
**HR**	.717							
**HI**	.433	.780						
**CI**	.252	.244	.761					
**RES**	.124	.203	.322	.758				
**TRU**	.020	.030	.218	.222	.755			
**OE**	.137	.084	.324	.311	.243	.790		
**KSI**	.152	.128	.253	.322	.237	.388	.843	
**SE**	.155	.200	.635	.216	.112	.201	.156	.863

*Note. The diagonal values in bold represent the square root of the AVE. The non-diagonal values represent the correlations among the latent variables.

**Table 5:** Fit statistics for the measurement/structural equation model.

Model	CMIN	CMIN/df	AGFI	TLI	CFI	GFI	RMSEA
Measurement Model	400.110	1.146	.914	.989	.990	.931	.020
Structural Equation Model	344.623	1.188	.919	.986	.987	.933	.023

### The structural model

After confirming the reliability and validity of the measurement model through confirmatory factor analysis, it is necessary to further analyze the model structure. Table 5 shows that the structural equation model has a good fitting degree: CMIN = 344.623; CMIN/df = 1.188; AGFI = 0.919; TLI = 0.986; CFI = 0.987; GFI = 0.933; RMSEA = 0.023.

**Fig 3:**
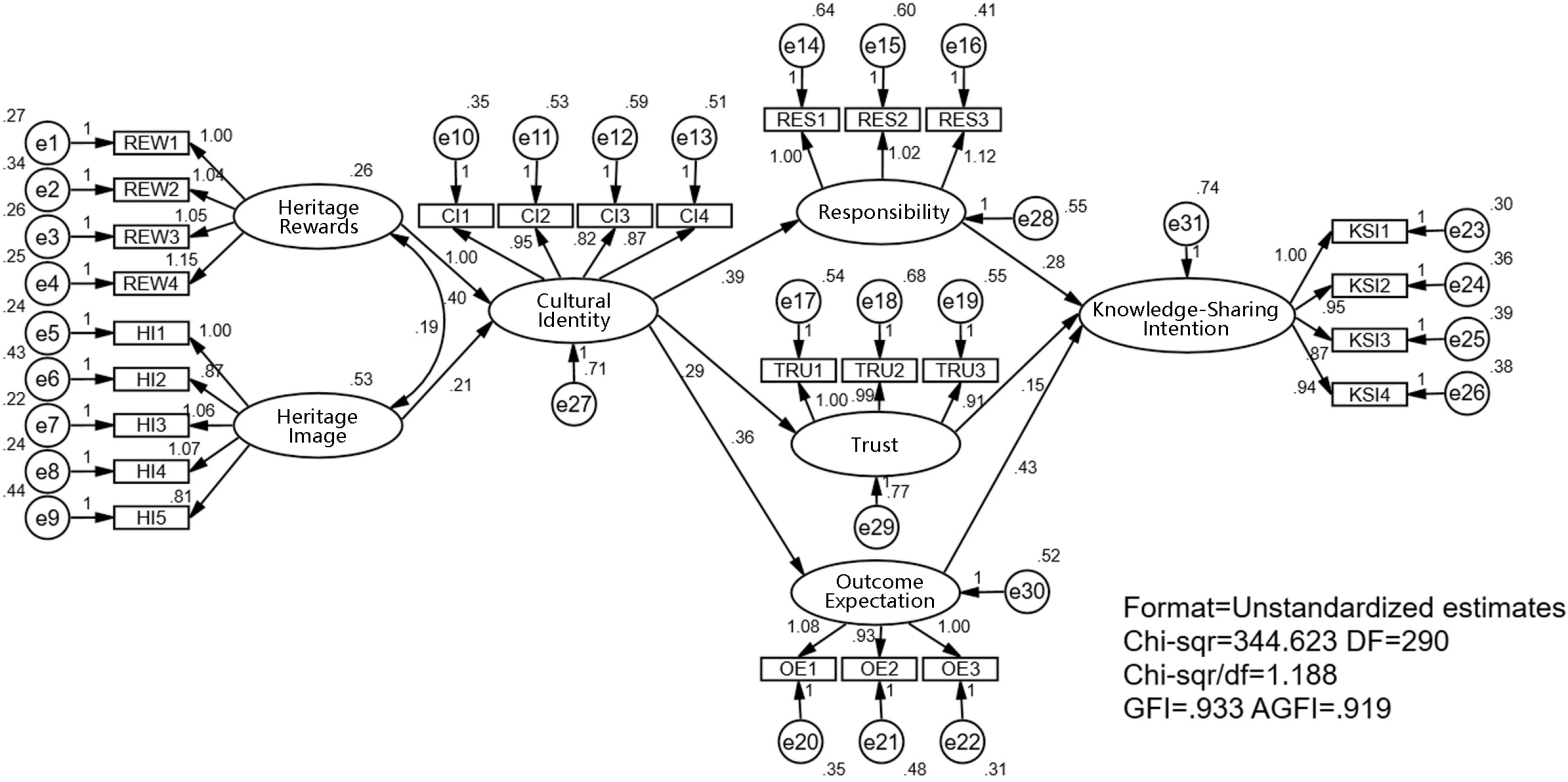
Path coefficient of the structural model.

The model path analysis results are shown in Table 6 and Fig 3. In the context of DRICH, heritage rewards have a significant positive effect on cultural identity (β = 0.230, SE = 0.131, p < 0.05), so H1 is supported. heritage image has a positive effect on cultural identity (β = 0.172, SE = 0.087, p < 0.05), and H2 is supported. Meanwhile, cultural identity has significant positive effects on outcome expectation (β = 0.409, SE = 0.054, p < 0.001), trust (β = 0.289, SE = 0.065, p < 0.001) and responsibility (β = 0.424, SE = 0.060, p < 0.001), among which outcome expectation has the strongest effect. Therefore, H3, H4, and H5 are all confirmed. Results also show that responsibility has a significant positive impact on knowledge sharing intention (β = 0.229, SE = 0.070, p < 0.001), trust has a positive impact on knowledge sharing intention (β = 0.139, SE = 0.061, p < 0.05), and outcome expectation has a significant positive impact on knowledge sharing intention (β = 0.342, SE = 0.061, P < 0.05). SE = 0.073, p < 0.001). Therefore, H6, H7 and H8 are confirmed.

**Table 6:** The results hypotheses test.

Hypotheses	Hypothesized path	B	β	S. E	t	Result
H1	HR → CI	0.404	0.230	0.131	3.093**	Supported
H2	HI → CI	0.213	0.172	0.087	2.454**	Supported
H3	CI → OE	0.360	0.409	0.054	6.621***	Supported
H4	CI → TRU	0.294	0.289	0.065	4.532***	Supported
H5	CI → RES	0.386	0.424	0.060	6.495***	Supported
H6	RES → KSI	0.275	0.229	0.070	3.904***	Supported
H7	TRU → KSI	0.149	0.139	0.061	2.431**	Supported
H8	OE → KSI	0.425	0.342	0.073	5.845***	Supported

Note: The symbol ‘***’ indicates a P value less than 0.01, ‘**’ indicates a P value less than 0.05 P-value test is ‘significant.’

**Fig 4:**
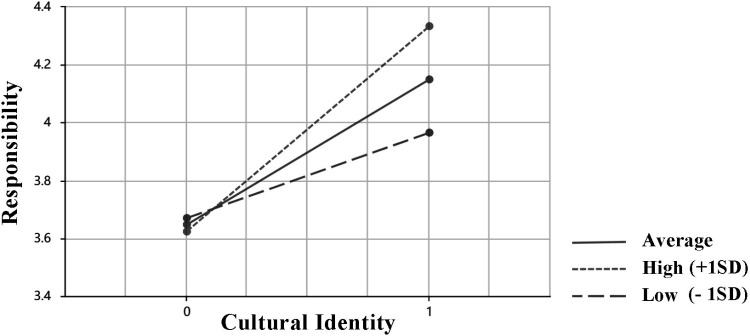
Simple slope plot of the moderating variable heritage-related self-efficacy (H5a).

In addition, this study further confirmed the moderating effect of SE. Fig. 4 shows the slope plot of SE as a moderating variable. The results show that SE positively moderates the relationship between cultural identity and responsibility (P < 0.05), and the effect size is 0.174. So H5a is confirmed.

## Discussion

This study applied SOR and SET assumption to investigate the effects of stimulus (Heritage rewards and heritage image) on organism (cultural identity, outcome expectation, trust and responsibility) and response (knowledge sharing intention) from the perspective of social exchange and measured the moderating effect of heritage-related self-efficacy. From the results of the study, all the research hypotheses are confirmed.

### Heritage rewards and heritage image can affect the organism

The SOR theory believes that a high level of external stimulus can improve an individual’s cognitive and behavioral influence, and this study further confirms this influence relationship.

Specifically, in the context of DRICH, the hypothesis that heritage rewards and heritage image as external stimulus affect organism feedback is supported in this study, which is similar to the conclusions of previous studies [149]. At the same time, the research results show the positive impact of heritage rewards on cultural identity (p < 0.05), which is consistent with the conclusion of Williams & Vaske (2003) [78]. Heritage image has a positive impact on cultural identity (p < 0.05), which is in line with the conclusion of Marcouyeux & Fleury-Bahi (2011) [150]. This implies that positive external stimulation plays a crucial role in enhancing knowledge sharing and fostering emotional and social connections related to DRICH. It stimulates public participation, sustaining long-term interest and enthusiasm, while deepening social identity and pride as cultural inheritors. Moreover, public participation is not solely driven by spontaneity but is influenced by various motivating factors that impact continued engagement. Heritage rewards and heritage image, as stimulus factors, can effectively influence organism feedback and guide individuals’ emotional states and behavioral expectations regarding the digitalization of intangible heritage. Moderate external stimulation not only enhances the initiative of individuals to participate, but also helps to cultivate their long-term participation enthusiasm and immersion, ensuring that individuals continue to pay attention to and contribute to this field.

### Outcome expectation, trust and responsibility can affect the response

The results of this study show that outcome expectation, trust, and responsibility are important variables in SET assumption. These factors have positive effects on the behavioral intention of individuals in the context of DRICH. First, outcome expectation has a significant impact on organism behavior, which is similar to the conclusions of Kankanhalli et al. (2005) [137], Chumg et al. (2015) [135], and Jin et al. (2015) [136]. The effect of outcome expectation on organism shows that it acts as a catalyst for individual internal motivation. This, in turn, motivates individuals to evaluate and choose the most appropriate behavior strategy more actively when facing different situations. It plays a shaping role in the path of action and decision-making processes taken by individuals to achieve established goals, and has a profound impact on dimensions such as emotional responses and social interactions. At the same time, compared with the two variables of trust and responsibility, the effect of outcome expectation on organism behavior is more significant. Second, research has shown the positive effects of trust on organism response, which is consistent with the study by Youssef et al. (2017) [74], and Rutten W, Blaas-Franken et al. (2016) [127]. The research also implies that in the context of DRICH, the establishment of trust is essential to reduce the uncertainty caused by information asymmetry. Once the public’s perception of trust in the intangible cultural heritage is established, it will also become an important driving force to stimulate their active participation and behavioral responses. This enhanced sense of trust not only helps the public to absorb and adopt relevant technologies more actively, but also further fuels their motivation for knowledge sharing, and promotes the innovation and development of agricultural cultural heritage. Finally, responsibility has a positive impact on organism behavior (response), which is similar to the result findings of Assiouras et al. (2019) [125], and Cannas et al. (2019) [121]. This means that by strengthening the guidance of public awareness of responsibility, organisms can be encouraged to show higher prudence in self-behavior, and the public can be motivated to participate in the protection, recording, dissemination and inheritance of intangible cultural heritage.

Based on the above findings, this study suggests that the public’s knowledge sharing in DRICH has its own complexity. High levels of outcome expectation, trust, and responsibility contribute to shaping a more inclusive and collaborative digital knowledge-sharing ecosystem. Individual and collective initiative, along with creativity in knowledge dissemination, cultural inheritance, and innovative practices, also play a significant role. Together, these factors affect the vitality and sustainability of DRICH and provide a supportive social foundation for its protection and development.

### The role of heritage-related self-efficacy

Research results show that heritage-related self-efficacy (SE), as a moderating variable, can positively moderate the relationship between responsibility and cultural identity (Fig 4), which is similar to the conclusion of Yang et al. (2022) [111]. In the context of DRICH, Individuals’ heritage-related self-efficacy (SE) can strengthen the positive relationship between cultural identity and responsibility. This reveals that the moderating effect of SE plays an irreplaceable role in the process of cultural identity affecting responsibility. Specifically, individuals with a high sense of SE are more likely to recognize their important role in cultural inheritance and motivate them to take proactive actions, which will also help improve public participation in activities of DRICH and lay a solid social foundation for heritage protection and inheritance. In addition, the study in three different groups, average, high and low, showed that SE has a sustained and significant impact on CI and RES. This means that at different levels of SE, the extent to which cultural identity influences responsibility may vary. A high level of SE can promote individuals’ confidence in their own abilities and significantly enhance the influence relationship between cultural identity and responsibility. However, with the decrease of SE, individuals’ motivation to participate in the digitalization of rural intangible cultural heritage (DRICH) activities will be reduced, and the positive dynamic relationship between cultural identity and responsibility will be weakened. SE provides an important perspective for this study to gain a more comprehensive and thorough insight into the far-reaching impact of cultural identity on the formation and development of individual responsibility.

## Implications

This study deeply analyzes the influencing factors and dissemination mechanism of agricultural digital intangible heritage, and has the following implications.

This study focuses on the knowledge sharing mechanism of intangible cultural heritage under the background of agricultural digitalization. Although previous studies have extensively discussed the dissemination mechanism of intangible cultural heritage [3] and technological innovation [20], the specific occurrence and influence mechanism of the knowledge sharing of agricultural digital intangible cultural heritage has been less discussed. This study integrates SOR and SET assumption to systematically analyze the interaction and influence mechanism of various variables in the knowledge sharing process of agricultural digital intangible heritage. This study has found that in the context of agricultural digital intangible cultural heritage, stimulus can motivate the public to participate in knowledge sharing of intangible cultural heritage by influencing their cognitive and emotional states. This interactive process constitutes the influence mechanism of agricultural digital intangible cultural heritage knowledge sharing and reveals how to promote the wide dissemination and living inheritance of intangible cultural heritage knowledge through effective external stimulation and active public participation. In addition, the study also emphasizes the necessity of constructing a multi-dimensional communication and protection strategy that takes multiple factors into account, including society, culture and technology. This strategy can not only promote the effective communication of rural intangible cultural heritage, but also enhance the public’s participation and protection awareness, and promote the sustainable development of ICH in rural areas.

Besides, this study has further explored the role of stimulus in the knowledge sharing intention of DRICH and applied the SOR theory to expand the application scenarios of this theory. Traditionally, studies on SOR mostly focus on analyzing the dynamics and behaviors of the consumer market in cultural heritage tourism [52,151] Based on the application scenario of DRICH, this study explores the dynamic evolution process of knowledge sharing from the perspective of social exchange combined with the SOR theory. The results show that in the context of DRICH, SET theoretical elements (cultural identity, outcome expectation, trust and responsibility) can participate as organismal feedback of the SOR theory to promote knowledge sharing behavior. The study demonstrates the applicability and effectiveness of this theory in explaining and predicting individual behavior in DRICH. Meanwhile, exploring in the application scenario of DRICH will also help to better understand the driving forces and potential obstacles of the knowledge sharing and dissemination. This cross-theoretical integration not only provides a new dimension for the application of the SOR theory, but also offers new strategies and perspectives for the knowledge dissemination and management of DRICH. The expansion of this application scenario enables researchers to study the knowledge management and dissemination from new perspectives and provides new theoretical support and practical guidance for related research and practice.

In addition, the role of SE should be emphasized in the protection of DRICH. In the context of agriculture, SE can improve individuals’ deep understanding of digitalization of intangible heritage. When individuals show a high degree of identification with the digital intangible cultural heritage, they are often motivated by an inherent sense of mission, which prompts them to take the initiative to share relevant knowledge, thus promoting the digital protection and inheritance of the intangible cultural heritage [111].

## Conclusion

Based on the SOR theory and SET assumption, this study develops a theoretical framework on the knowledge sharing intention of DRICH. Through empirical analysis, it is confirmed that reward and heritage image can affect organism feedback, and then drive individual behavior intention of knowledge sharing. At the same time, this study has further verified the validity of SET in explaining individual behavioral intention and the moderating effect of heritage-related self-efficacy on organismal feedback. In conclusion, this study provides a new perspective for the protection and inheritance of DRICH, deepens individuals’ understanding of the knowledge sharing and behavioral motivation, improves the public’s transmission. Additionally, this study strengthens the cultural confidence of rural community members [3]. The study also provides important theoretical basis for policy makers and practitioners, and sheds light on the establishment of effective communication incentive strategies and related research.

It should be noted that there is still room for improvement in this study. As this study applies quantitative research methods, it fails to adequately account for the wide diversity and deep complexity of participants. The follow-up research can further collect qualitative data (such as interviews), and adopt a combination of quantitative and qualitative methods to cross-verify the content and conclusions of this research, so as to improve the effectiveness of the research. Meanwhile, the sample of this study may not be sufficiently representative of the population, which restricts the universal applicability of the conclusion drawn in this study. To address this limitation, it is necessary to collect and use more extensive and diversified samples in future research. In addition, stratified sampling or multistage sampling strategies should be executed for the improved representativeness and universality of research results. Finally, this study did not fully refine the classification of DRICH, as well as the uniqueness of the attributes of various types and their impact on individual behavior. Future research can conduct in-depth exploration into the specific characteristics of DRICH in its internal categories and deepen the validity and practicability of this research.

## Supporting information

S1 File Raw image. (PDF)
