# Supplementary material for: Exploring knowledge sharing intention of digitalization of rural intangible cultural heritage (DRICH): Integrating stimulus-organism-response (SOR) theory and social exchange theory (SET)
Source: PLoS One. 2025 Jun 13;20(6):e0325892. doi: 10.1371/journal.pone.0325892 (PMC12165400; doi:10.1371/journal.pone.0325892)
Supplement: S1 File — (PDF) [file pone.0325892.s001.pdf]

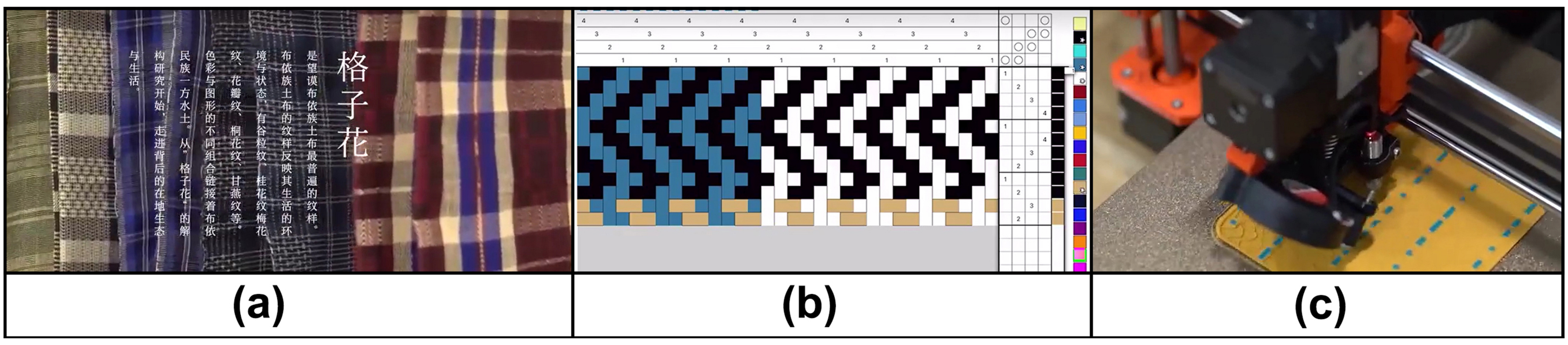

Fig 1. Digitalization of traditional clothing pattern conversion on xiaohongshu platform

(a) Traditional checkered patterns (b) Digital coding and translation of checkered pattern

(c) Digital model-based execution of printing and weaving

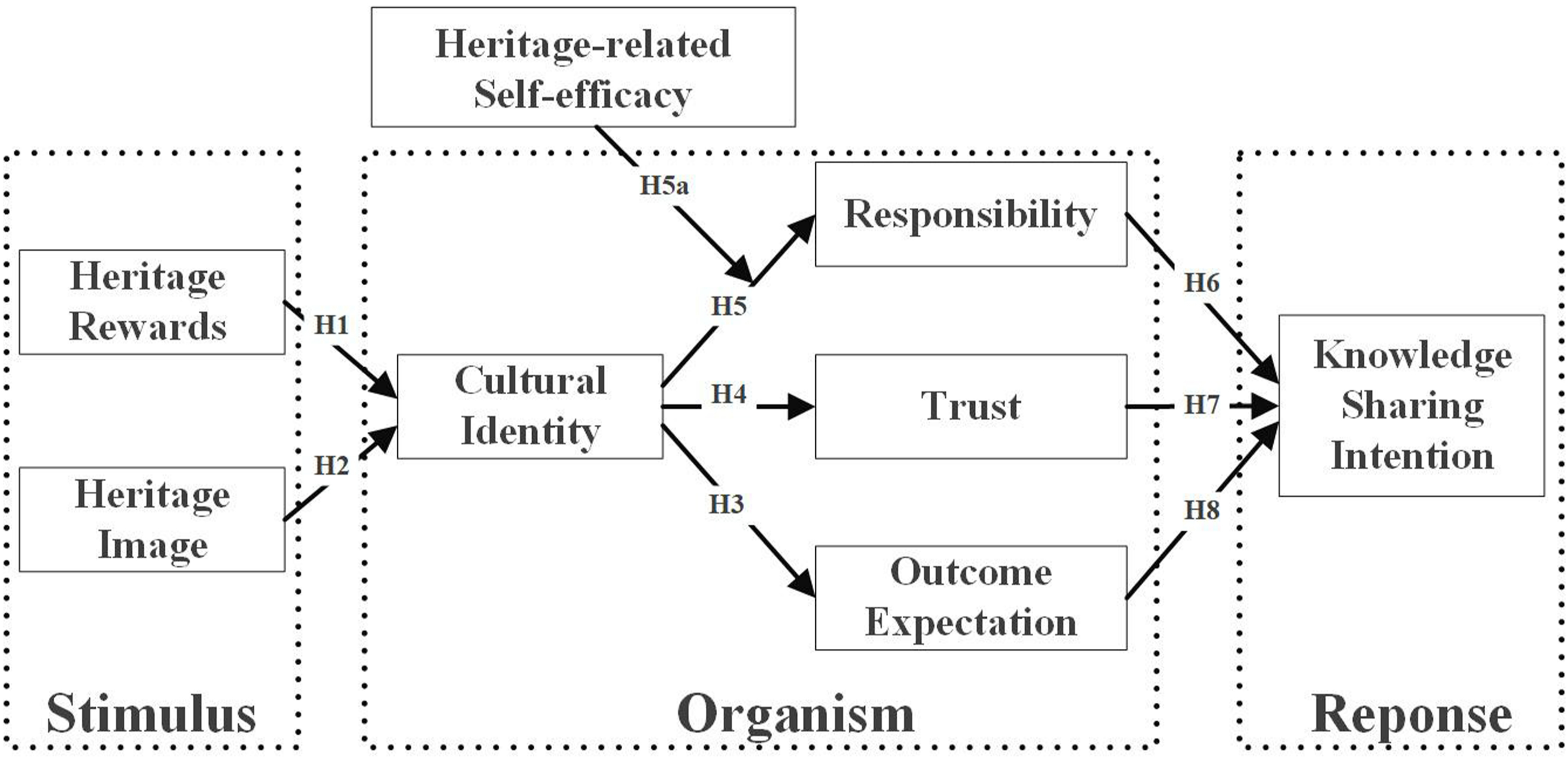

Fig 2. The conceptual framework of the research

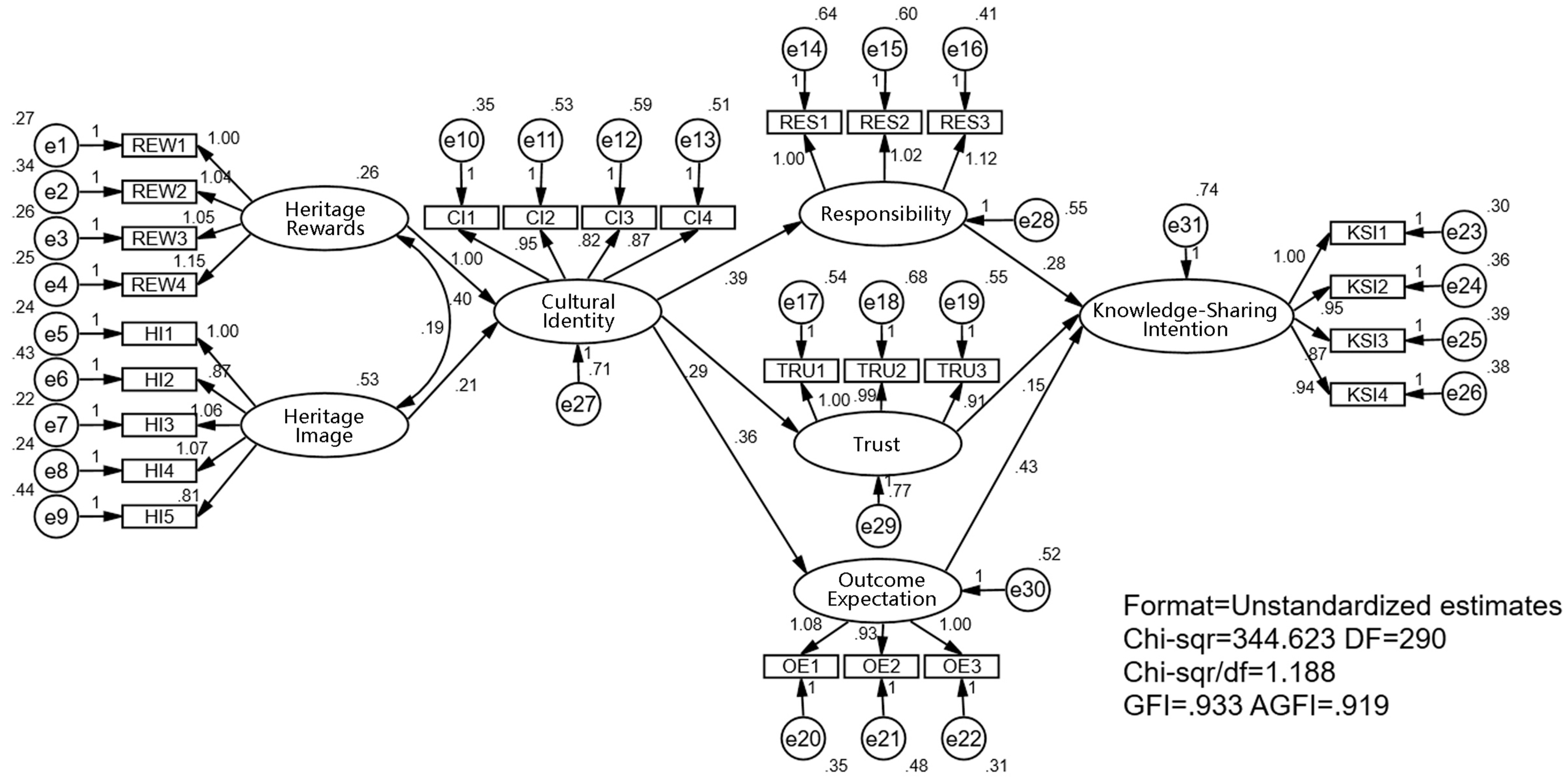

Fig 3. Path coefficient of the structural model

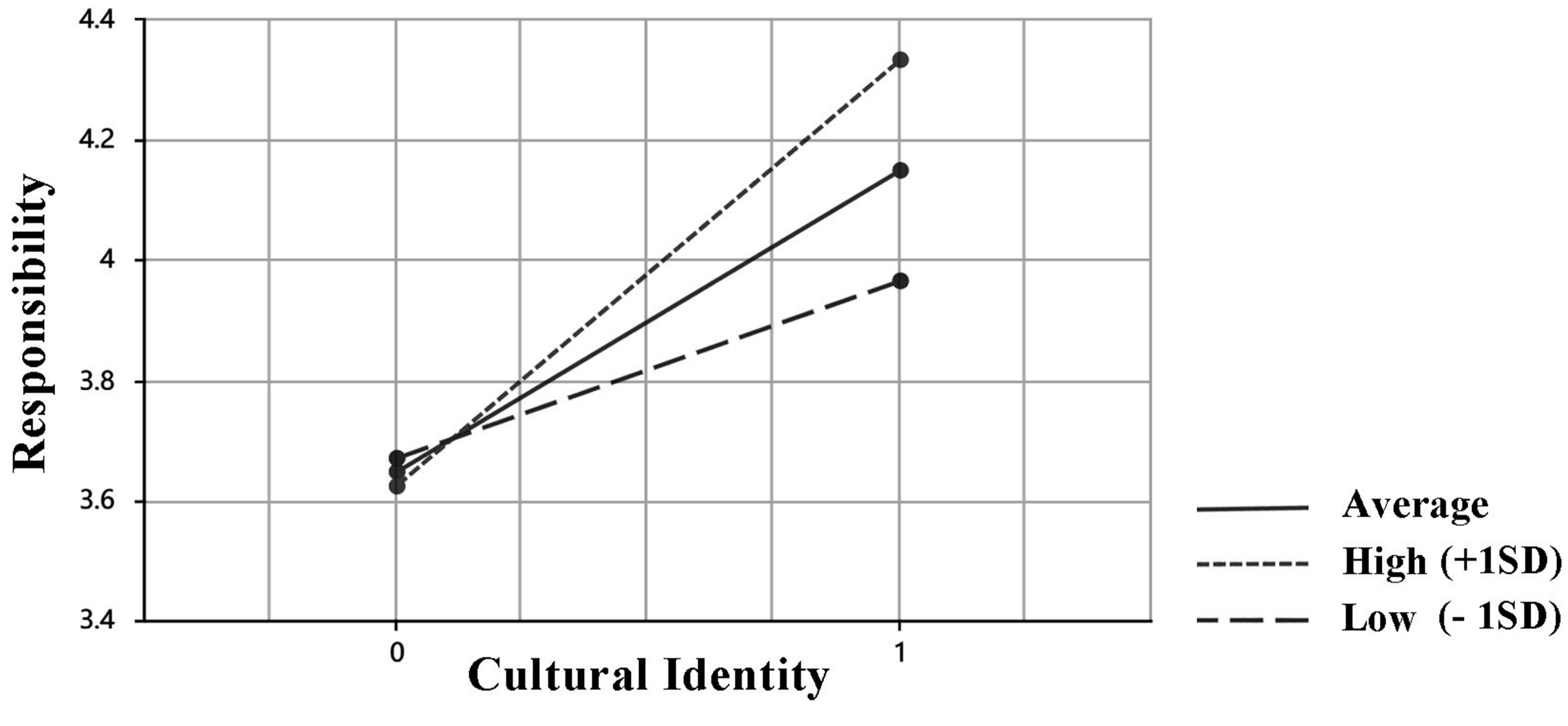

Fig 4. Simple slope plot of the moderating variable heritage-related self-efficacy（H5a）
